# Overlooked Complications and Opportunities in the Development of Drugs Based upon Macrobicyclic Peptides: The “Homeomorphic Switch”

**DOI:** 10.1021/jacs.5c21032

**Published:** 2026-02-26

**Authors:** Isabelle J. Smith, Simon M. Popovic, John A. Gladysz

**Affiliations:** Department of Chemistry, Texas A&M University, PO Box 30012, College Station, Texas 77842-3012, United States

## Abstract

Many macrobicyclic compounds can undergo a widely overlooked conformational process, homeomorphic isomerization, that effectively turns molecules inside-out and leads to species with *in,in*, *out,out*, *in,out*, and *out,in* bridgeheads. Different chemical and biological properties would be expected. Some foundational aspects of this phenomenon are briefly reviewed, with both model compounds of the formula E­((CH_2_)_*n*_)_3_E (*n* ≥ 10, E = P, As, etc.) and macrobicyclic peptides. Attempts are made to bridge the differing cultural perspectives of the physical organic and chemical biology communities regarding bicyclic molecules. Opportunities are presented for (1) improving the stereochemical definition of macrobicyclic peptides and their adducts with proteins, (2) new drug design and efficacy protocols, and (3) refining or circumventing existing patented IP.

## Graphical abstract

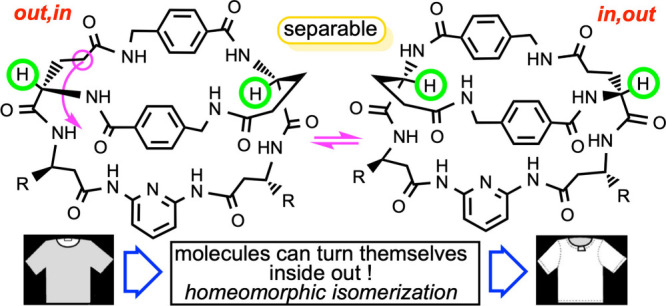

## 1 Introduction

### 1.1 Peptide Conformations

From a conformational standpoint, peptides are one of the most studied classes of organic molecules.^1^ As is well known, the appreciable nitrogen/carbon multiple bonding in the amide linkages (−(H)**N** ^...^ **C**(^...^O)–CH­(R)−) gives rise to *Z*/*E* isomers.^2^ Furthermore, appropriate sequences of amino acid residues give rise to a variety of special motifs, such as alpha helices, beta sheets, beta turns, omega loops, and many others.^1^ All of these bring hydrogen bonding into play.^1,2^ Together, they represent major determinants of protein structures.

In some cases these conformational motifs can interconvert, for example via “unfolding”, a process in which hydrogen bonds are disrupted. Alternative protein conformations can be benign, but when certain isomers are generated *in vivo*, prion diseases and protein misfolding disorders can result.^3^ In humans, these are commonly progressive, incurable, and fatal neurodegenerative conditions. Some of the little-known conformational processes depicted in the graphics below have not, to the authors’ knowledge, been previously considered as possible contributors. And as detailed in the following text, these processes can manifest themselves in the title macrobicyclic peptides, some of which themselves are being pursued as drug candidates for prion diseases.^4^

### 1.2 Peptide Drugs

There is intense current interest in the development of peptide drugs, particularly those that are orally administrable.^5^ There are numerous practical challenges with oral delivery,^6,7^ and in 2022 nearly 90% of peptide drugs were administered intravenously.^6^ One challenge is posed by the many digestive enzymes found in the gastrointestinal tract (e.g., peptidases such as chymotrypsin, carboxypeptidases).^8^ Another is to penetrate the entangled oligomeric glycoproteins (gel-forming mucins) that line the gastrointestinal tract and protect the underlying epithelia.^9^ Finally, the drug must navigate a paracellular absorption barrier into the epithelia.^10^

For a variety of well-reviewed reasons, macrocyclic peptides have featured prominently in drug development over the last quarter century.^11^ One impetus is that the restricted range of conformations vis-à-vis acyclic peptides may better promote interactions with cellular targets. For still additional reasons, macro *bi* cyclic peptides have exploded onto the drug development scene in the past decade,^12^ and a number of startups have sought to exploit them. Such peptides can exhibit several special but poorly recognized conformational and dynamic properties, as illustrated with simpler classes of bicyclic molecules below, and further developed in several recent articles from other laboratories that are treated in the penultimate section.^13^ The goal of this Perspective is to highlight overlooked commonalities that have implications for biological properties, medicinal applications, and the protection of intellectual property. However, foundational concepts need to be established first.

## 2 Bicyclic and Polycyclic Molecules: Conventions

Textbooks generally sidestep rigorous definitions of bicyclic, tricyclic, and higher polycyclic molecules. As an undergraduate, one author was taught to use the “scissors test”. As exemplified in Figure 1 (top), a bicyclic molecule is rendered acyclic with a minimum of two cuts, a tricyclic molecule with three, and so forth. In most cases, the cuts can be implemented at multiple sites.

**Figure 1:**
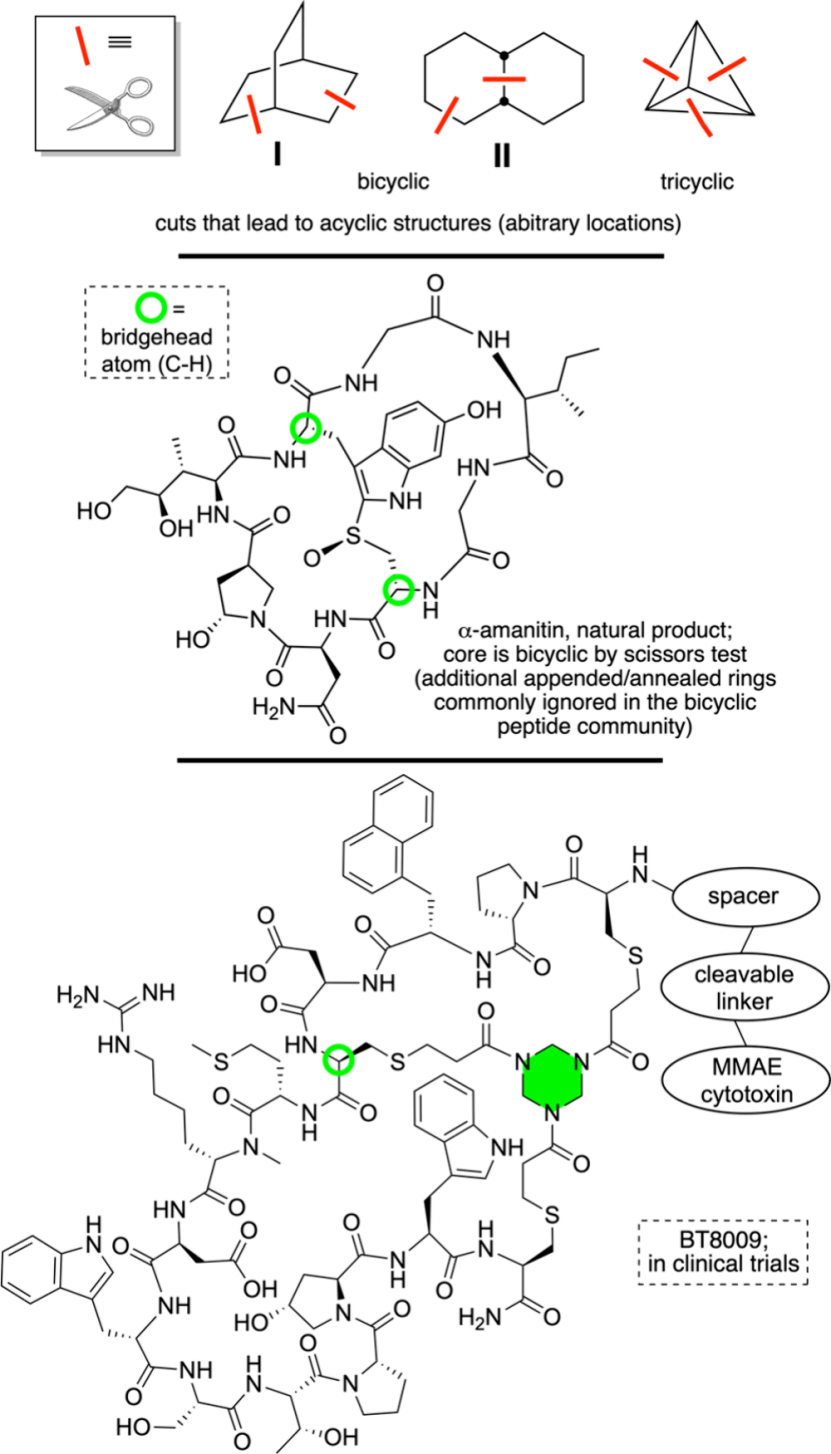
One approach to defining the number of rings in polycyclic molecules (top) and representative peptides generally considered to be macrobicyclic.

However, what about cyclic groups attached to the core structure (e.g., a phenyl ring) or annealed onto one of the bridges or tethers? Several organic colleagues were polled, and most thought they should be counted also. However, the cyclic peptide community ignores these, and would view α-amanitin in Figure 1 as bicyclic (as opposed to pentacyclic including the 6- and two 5-membered rings). The same would hold for the more complex structure BT8009 (Figure 1), currently the focus of a >900-person clinical trial for the treatment of advanced urothelial cancer.^14^

In traditional bicyclic molecules, there are two bridgehead atoms and three bridges or tethers, as illustrated for bicyclo[2.2.2]­octane (**I**) and bicyclo[4.4.0]­decane (**II**) in Figure 1. Compound **II** is arbitrarily rendered as the *cis* isomer (*cis*-decalin) as opposed to *trans*. Thus, it is obvious from the get-go that bicyclic molecules can exist as bridgehead-based stereoisomers. Beyond these fundamentals, the additional feature emphasized in this Perspective—recognized by only a few within the peptide community^13b–13d^ —is that when the tethers are long and flexible enough, such molecules can turn themselves inside-out.

## 3 Macrobicyclic Compounds and Homeomorphic Isomerization

### 3.1 General Aspects

The bridgehead atoms of bicyclic organic compounds may be tetravalent, as in CH, CR, SiR, or P=O groups, or trivalent, such as nitrogen (N**:**) or phosphorus (P**:**) atoms. As shown in Figure 2 with E–X as a general bridgehead representation, there are three limiting structures, **III** (termed *in,in*), **V** (*out,out*), and **VII** (*in,out*).^15,16^ When the upper and lower hemispheres of the *in,in* and *out,out* isomers are identical, the *in,out* and *out,in* isomers are degenerate. Only a few bicyclic molecules adopt conformations with rigorously linear X–E···E–X axes. Hence, as shown in **IX** (Figure 2), an *out* bridgehead can be defined as one with an E···E–X angle of 180–90° and an *in* bridgehead as one with an E···E–X angle of 0–90°.

**Figure 2:**
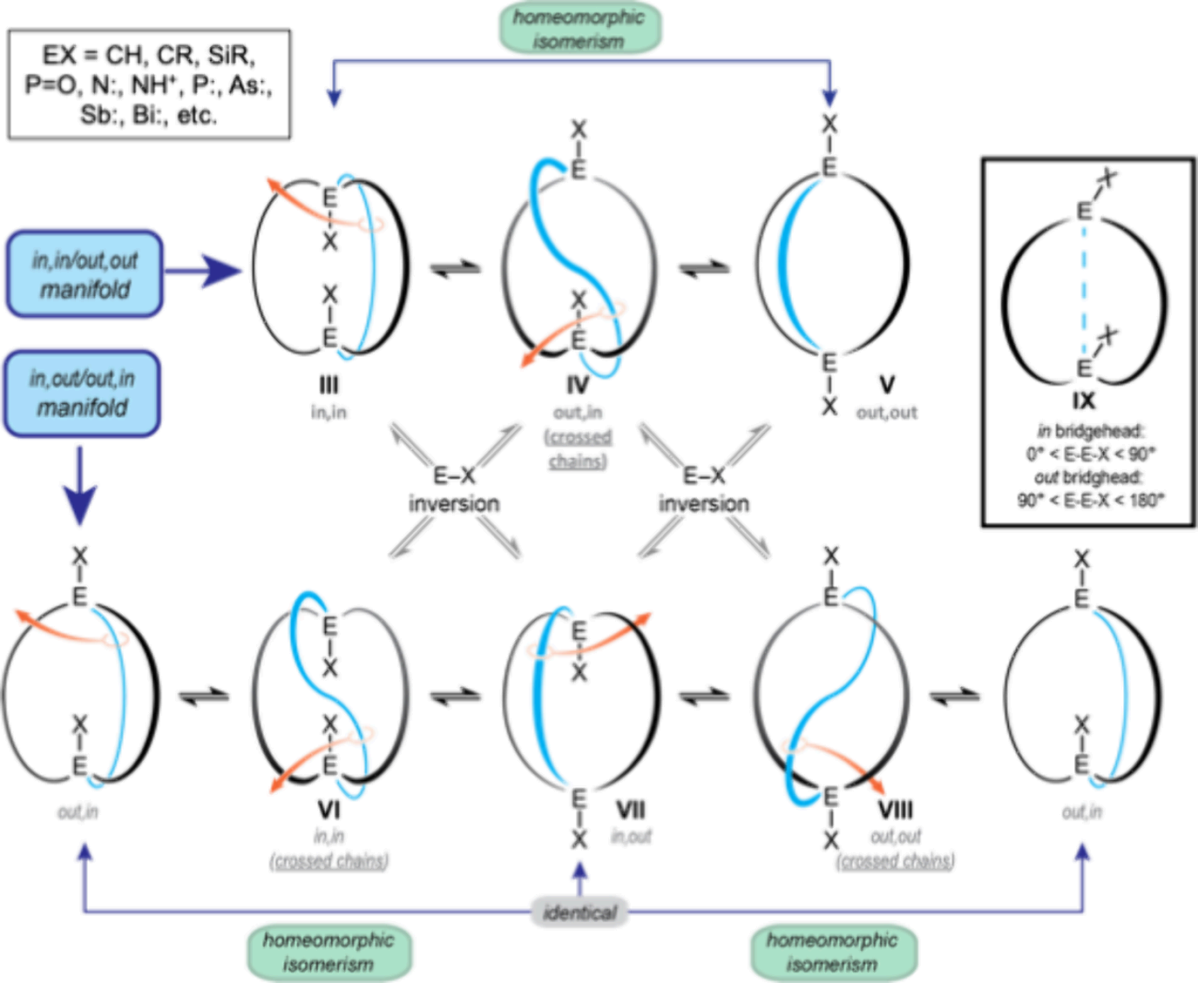
Homeomorphic isomerizations of macrobicyclic molecules and a geometric test for *in* and *out* bridgeheads (**IX**).

When most chemists consider a potential equilibrium between *in,in* and *out,out* isomers (e.g., **III** ⇆ **V**), they would probably first deem an inversion of configuration at each bridgehead atom to be necessary (a pyramidal inversion in the cases of nitrogen or phosphorus). Ditto for the *in,out* and *out,in* isomers. These would be extremely high energy processes for compounds with CH, CR, SiR, or P=O bridgeheads. In contrast, inversion would be rapid at room temperature for nitrogen (as found in ammonia), and require temperatures of ∼150 °C for phosphorus.^17^

However, when the tethers connecting the bridgeheads are sufficiently long and flexible, there is a topological process that interconverts the *in,in* and *out,out* isomers (or *in*,*out* and *out*,*in*). This was termed homeomorphic isomerization by Howard E. Simmons in 1968, during the course of his studies of compounds with N**:** and NH^+^ bridgeheads at DuPont.^15^ The expression is rigorously correct from a topologist’s perspective. This transformation can be effected by “grabbing” one tether through the macrocycle formed by the other two and pulling it through the macrocycle (**III** ⇆ **IV** ⇆ **V** in Figure 2). A video of this motion is excerpted in Figure 3.^18^ The process is tantamount to turning the molecule inside-out.

**Figure 3:**
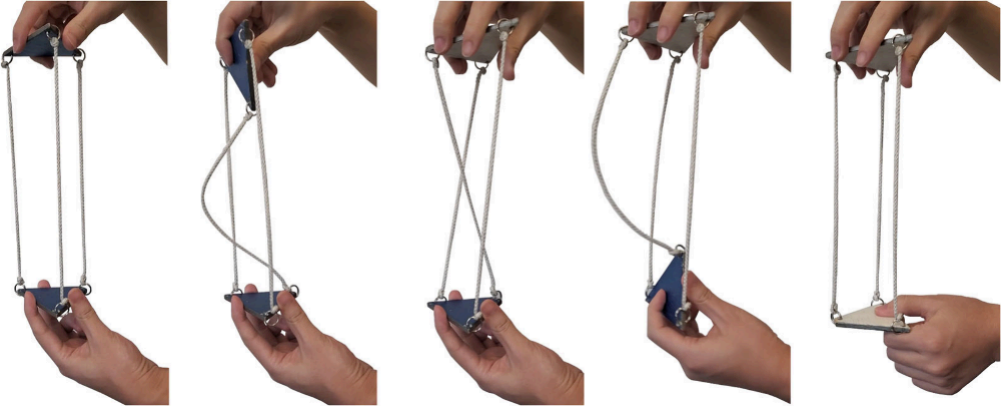
Visualization of homeomorphic isomerizations with models in which the triangles represent pyramidal bridgehead atoms or ring. The blue-colored sides are exchanged with white-colored sides.

Figure 2 also illustrates additional aspects of these isomerizations. In the upper *in,in*/*out,out* manifold, there is an intermediate “cross chain” species (**IV**), also evident in the middle panel in Figure 3. In the lower *in,out*/*out,in* manifold, there are two distinct cross chain species, one with both E–X bridgeheads *in* (**VI**) and the other with both *out* (**VIII**). In one case to date,^19^ it has been possible to render **VIII** more stable than **VII** (uncrossed) by making the X groups very bulky.^20^ Figure 2 also shows how structures in the upper *in,in*/*out,out* manifold can equilibrate with those in the lower *in,out*/*out,in* manifold by inversion of a single E–X configuration.

### 3.2 Specific Molecules

Figure 4 (bottom) depicts data from the authors’ laboratory involving trivalent phosphorus (P**:**) bridgeheads tethered by methylene segments of equal lengths, (CH_2_)_*n*_ (*n* = 10, 12, 14, 16, 18).^18,21^ At first glance, the systems **X**–**XIII** may seem far removed from biological relevance. However, their fundamental properties (and those of arsenic analogs below)^22^ have special importance in view of exciting applications of newly synthesized macrobicyclic peptides that have bismuth, arsenic, and nitrogen bridgeheads.^23^ Representatives of various families are depicted in Figure 5 and treated further below.

**Figure 4:**
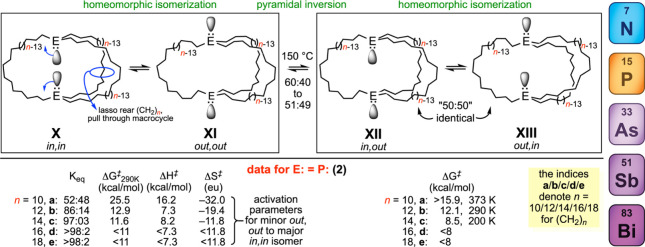
Homeomorphic isomerizations investigated in the authors’ laboratory.

**Figure 5:**
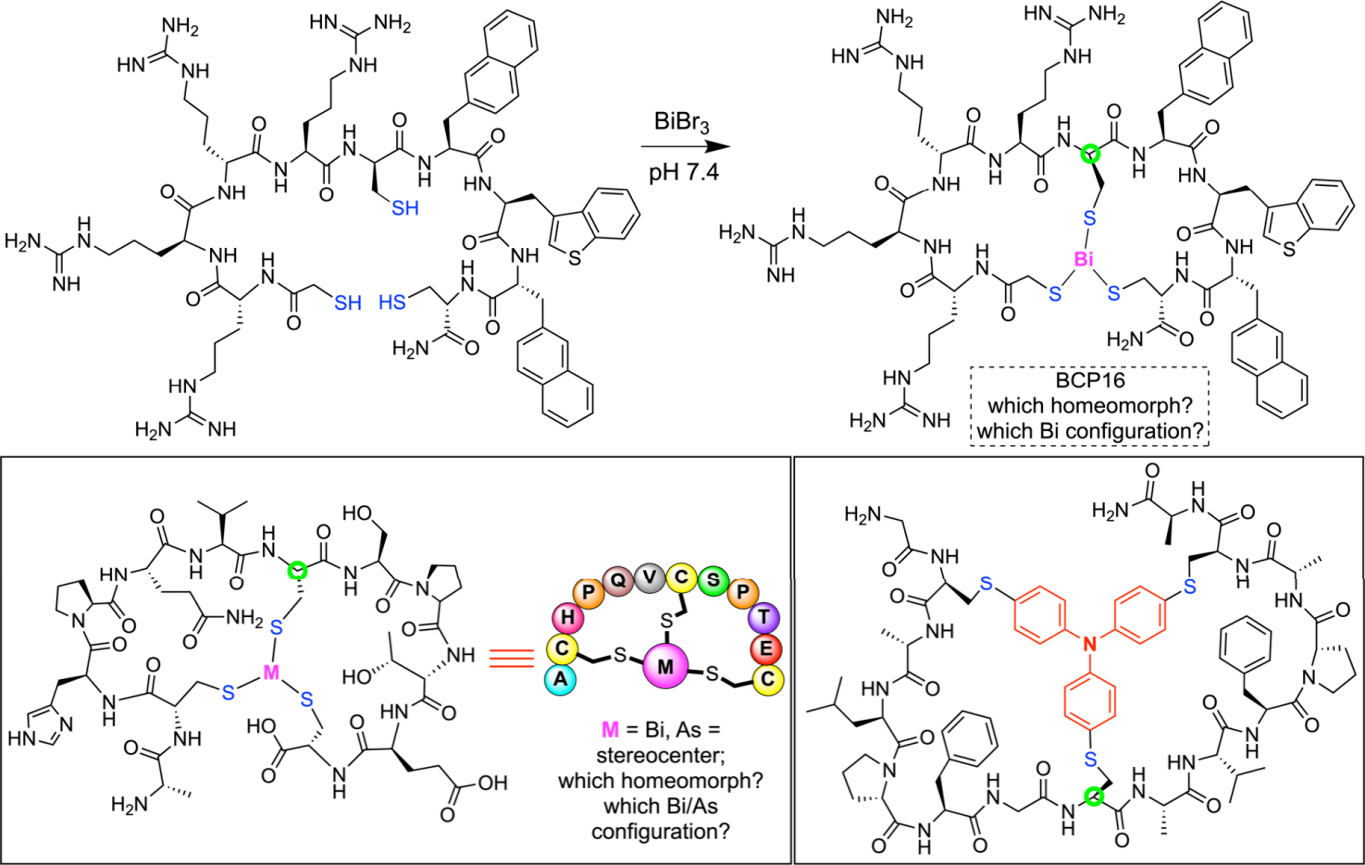
Macrobicyclic peptides with bismuth,^23b,23c^ arsenic,^23b^ and nitrogen^23a^ bridgeheads.

Consistent with much literature precedent,^17^ the dibridgehead diphosphines in Figure 4 undergo pyramidal inversion only at 150 °C (left versus right boxes). However, with (CH_2_)_*n*_ tether lengths of 10 or more, homeomorphic isomerization occurs at room temperature (albeit slowly for *n* = 10). In all cases, the activation energy increases as the macrocycle size or tether length decreases. Also, the entropy of activation becomes more negative at shorter tether lengths, as more degrees of freedom are sacrificed during the “pull through”. Due to lone pair/lone pair repulsion and other factors, the *in,in* isomer **X** should become increasingly destabilized as the tether length further decreases below *n* = 10.

Either prior to or after the authors’ work in Figure 4, there have been ca. six other explicit demonstrations of homeomorphic isomerization with different types of molecules.^24–26^ “Explicit” means that (1) both *in*,*in* and *out*,*out* (or non-degenerate *in*,*out* and *out*,*in*) isomers can be observed, and a direct path can be established between them (e.g., appropriate NMR experiments), or (2) non-equivalent bridgehead E–X moieties of degenerate *in,out* and *out,in* isomers can be observed and their exchange established. However, the phenomenon is undoubtedly widespread, as there should be numerous macrobicyclic molecules capable of homeomorphic isomerization at room temperature, but with rates so rapid that the two forms are challenging to independently observe.^27^

An elegant study of bicyclo[6.5.1]­tetradecane (**1**, Figure 6) has shown that homeomorphic isomerization is possible even in medium sized rings with one very short tether—in this case, a single methylene group.^25,28^ The *in*,*in* and *out*,*out* isomers equilibrate at 60–90 °C with an Arrhenius activation energy of 24 kcal/mol (log A = 15.5). Another study involving separable but interconvertible *in,out* and *out,in* isomers of a macrobicyclic peptide-like molecule^26^ is presented below.

**Figure 6:**
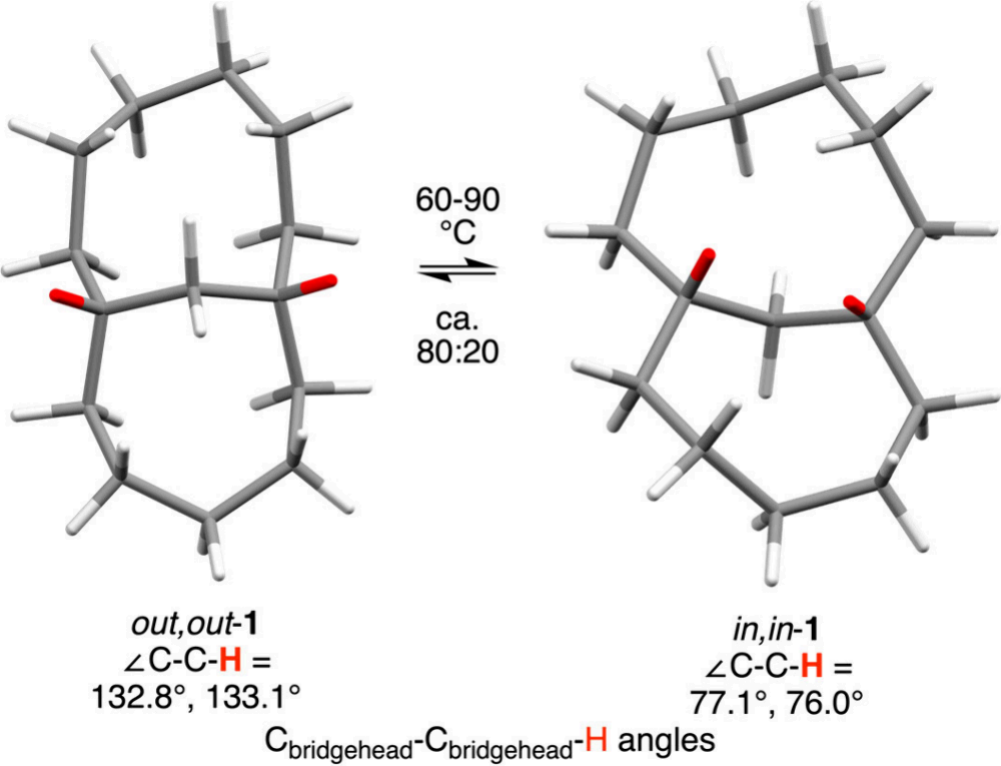
Homeomorphic isomerization of a bicyclic hydrocarbon with a single atom tether. The bridgehead hydrogen atoms are highlighted in red.

All macrobicyclic peptides will be chiral and the bridgehead atoms will normally be stereocenters. As depicted in Figures s1 and s2 in the Supporting Information (SI), homeomorphic isomerization does not invert the absolute configurations of such bridgehead atoms. As is intuitive given the absence of bond-breaking, an atom with the Cahn–Ingold–Prelog designation *R* remains *R*, and *S* remains *S*. Only in the case of a pyramidal inversion, as would commonly be facile with trivalent nitrogen (N:) bridgeheads, would *R* and *S* configurations be exchanged.

It is instructive to return to Figure 5 in this context. The bridgeheads in all three bicyclic structures depicted are the SCH_2_ **C** H­(C=O) carbon atoms of “interior” cysteine residues (green circles) and either (1) pyramidal bismuth or arsenic stereocenters that have prohibitively high inversion barriers^17^ or (2) nitrogen stereocenters that likely undergo rapid inversion at room temperature. To the authors’ knowledge, the absolute configurations of the bismuth and arsenic stereocenters (*R* or *S*) have never been specified in the open^23^ or patent^29^ literature for this growing class of compounds. The syntheses may have delivered mixtures or been diastereo­selective. In such situations, sometimes there is a steric rationale as to why one diastereomer might be favored over the other, but as Figure s3 in the SI shows, there is no *a priori* reason to expect a significant difference. And beyond this point, homeomorphic isomerization remains possible, for example with a *R*_Bi_ bridgehead remaining *R*_Bi_, an *S*_Bi_ bridgehead remaining *S*_Bi_, etc.

## 4 Inside-Out Objects and Molecules: Some Potential Uses

There are a variety of consumer products that have added value because they can be turned inside-out. For example, a T-shirt can be worn either way, with the seams and label out often representing a fashion statement (or a severe hangover). Some shirts or sweaters are designed to display two different external colors, as are all-in-one home-and-away basketball jerseys. The most probable such item to be found in a reader’s closet would be a reversible jacket, perhaps water-repellant on one side and more absorbent on the other, as illustrated in Figure 7.

**Figure 7:**
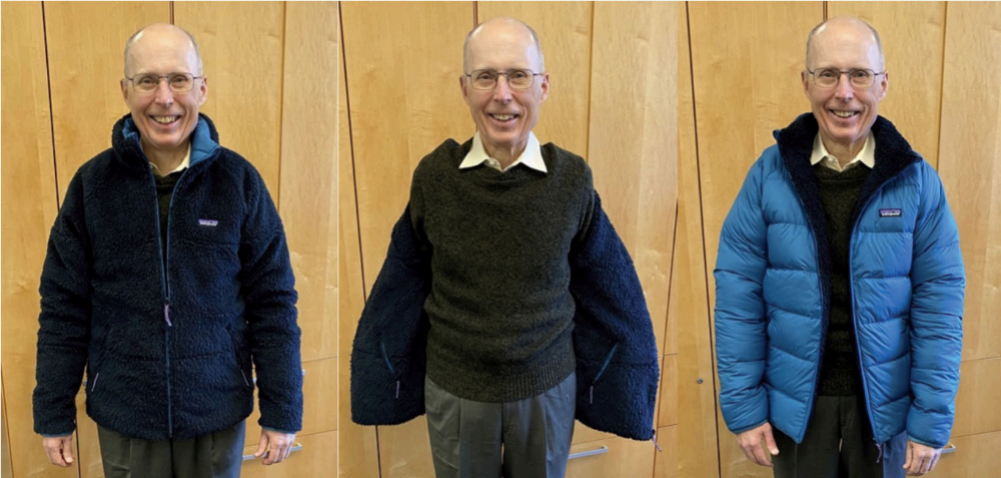
Homeomorphic isomerization illustrated with a reversible jacket.

The authors have shown that the dibridgehead diphosphines in Figure 4 reversibly bind certain metal fragments, and can be used as “container molecules” for transport purposes.^30^ The underlying chemistry for one case is illustrated in Figure 8 (top).^18^ The diphosphine **2c**, which has 14-carbon (CH_2_)_14_ tethers, exists as a rapidly equilibrating 97:03 mixture of *in,in* and *out,out* isomers per the activation parameters in Figure 4. Additions of PtCl_2_ afford the cage-like platinum complex **3c**. Various data suggest that the minor *out,out* form first binds to platinum (as in **4c**), which is delivered into the chelate cage by a homeomorphic isomerization. In the presence of excess potassium cyanide, the platinum is extruded by a similar process, driven by the higher “formation constants” associated with Pt-CN adducts.

**Figure 8:**
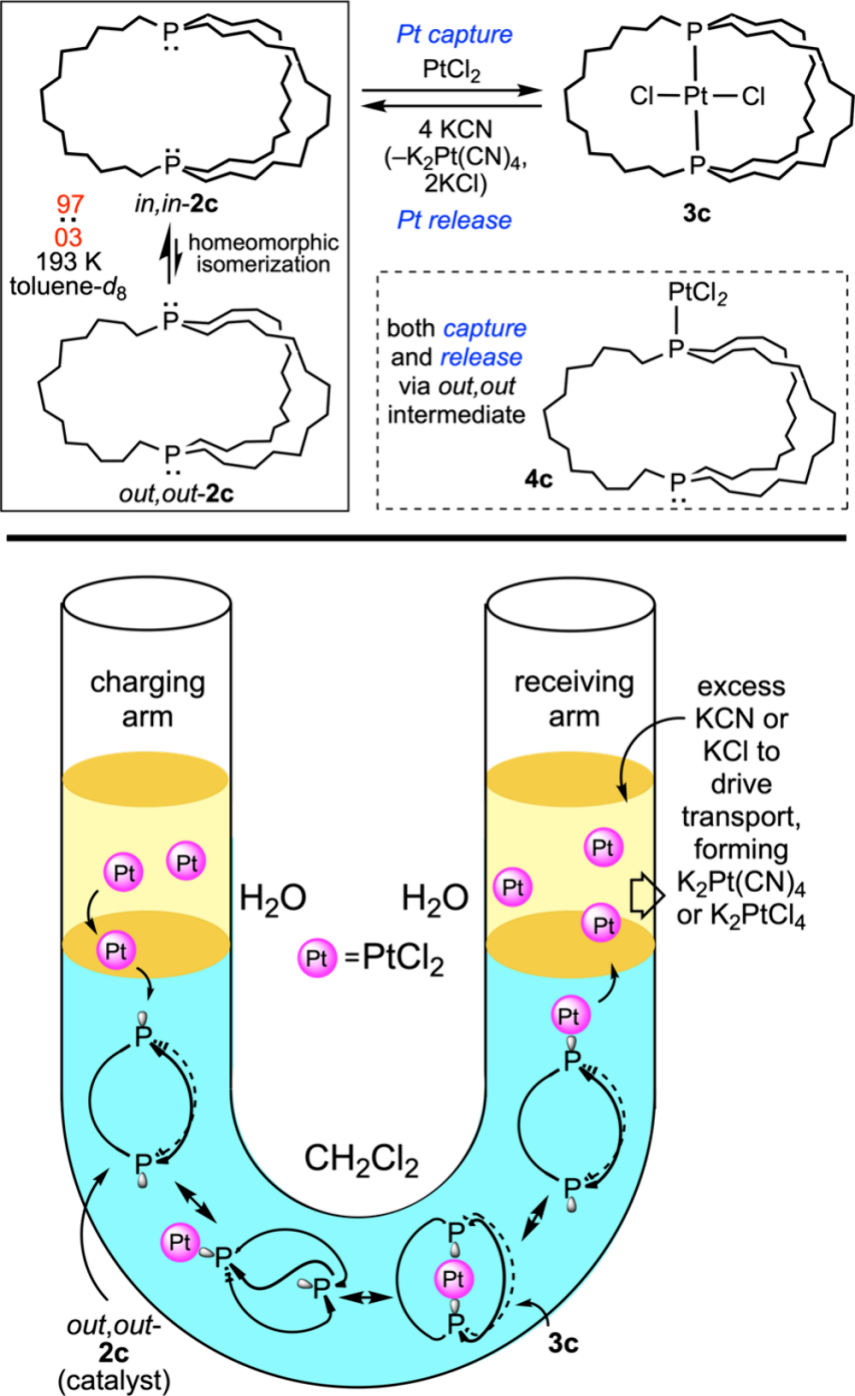
Capture and release of a PtCl_2_ payload by a dibridgehead diphosphine capable of homeomorphic isomerization.

These phenomena can be exploited for U-tube separations as shown in a minimalist cartoon in Figure 8 (bottom).^30^ When such experiments are carried out with mixtures of PtCl_2_, PdCl_2_, and NiCl_2_, palladium is transported from the charging arm to the receiving arm slightly faster than platinum, and nickel does not move at all. The dibridgehead diphosphine **2e** with longer (CH_2_)_18_ tethers functions similarly, but a host of other diphosphines and monophosphines are ineffective. Extensions to f-block metal separations are being pursued with the corresponding diphosphine dioxides.

At first glance, the phenomena in Figure 8 might seem to have little to do with macrobicyclic peptides. However, there are many well documented cases of peptide ionophores that bind metals or other cations (e.g., ammonium salts) that play roles in biological processes. The gramicidins, a family of *acyclic* pentadecapeptide antibiotics that see widespread clinical use, represent prominent examples.^31^ Gramicidins exhibit both non-channel and helical channel conformations in membranes, with only the latter capable of ion transport. The conformations of a family of immunosuppressive monocyclic macrocyclic undecapeptides, the cyclosporins, are very sensitive to the presence of metal ions (and many other factors).^2^ There is a less extensive but growing literature of cation binding to macrobicyclic peptides.^32^ Thus, it would be easy to envision that one homeomorph of a macrobicyclic peptide might have a very high affinity for a certain biological cation, and the other a very low affinity.

## 5 Homeomorphism: Additional Motifs

The following systems are relevant to some of the macrobicyclic peptides attracting current interest. First, consider two rings connected by three tethers, such as **XV** in Figure 9 (top). Note that by the “scissors test” (Figure 1) this would be a tetracycle, but following usage in the peptide community, the authors continue to apply the term macrobicycle. In any case, when one tether is pulled through the macrocycle defined by the other two, the *in* and *out* faces of each ring are exchanged. This process would be degenerate when the “bridgehead rings” are symmetrically 1,3,5-trisubstituted benzenes, but if the faces were differentiated (e.g., by π complexation of a metal), the two forms would be inequivalent.

**Figure 9:**
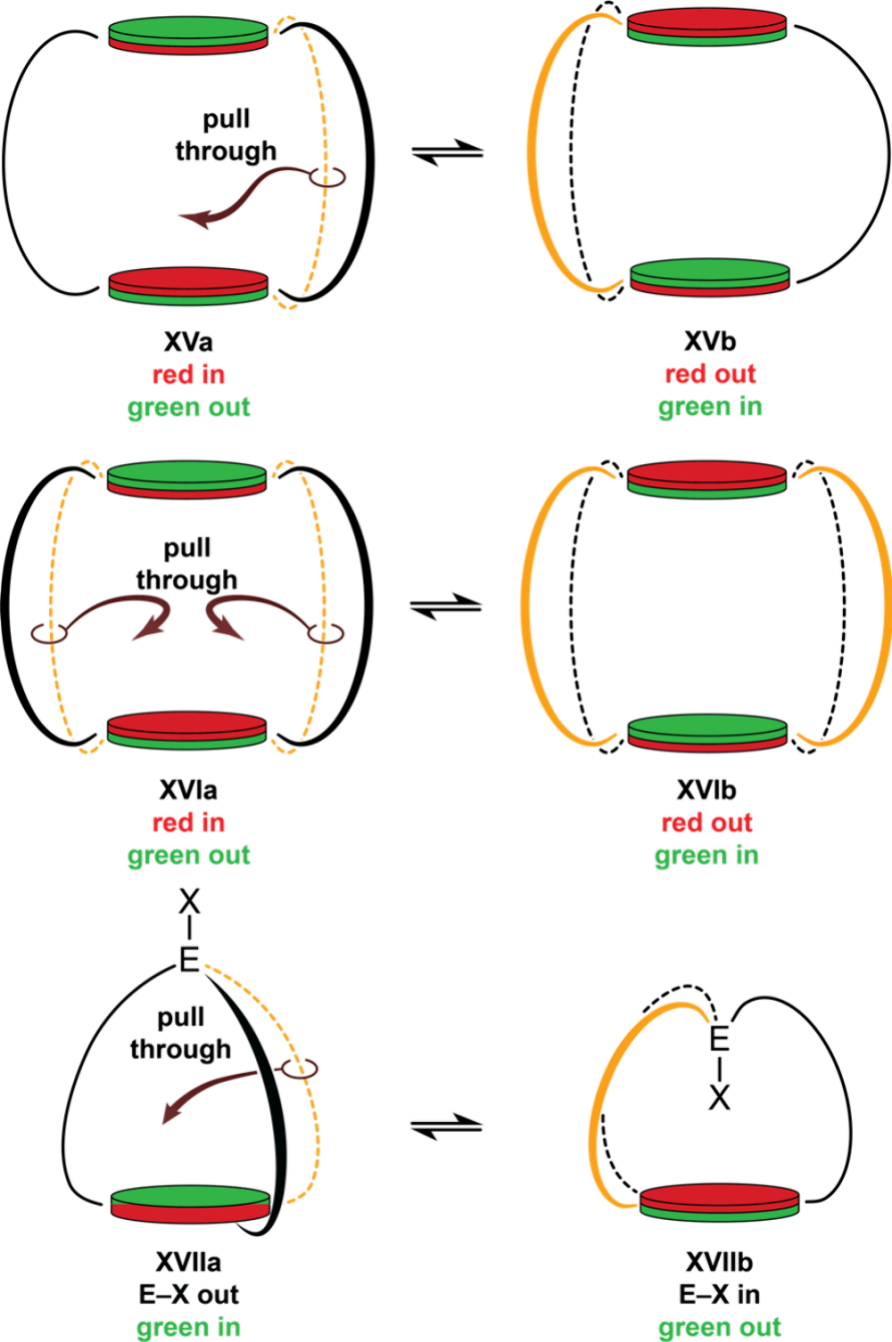
Homeomorphic isomerization involving molecules with “bridgehead rings” in place of bridgehead atoms and/or four or more linkages between the bridgeheads.

In principle, compounds in which bridgehead rings that are connected by four or more tethers can also undergo homeomorphic isomerization. As shown in **XVI**, one simply pulls all the tethers through the macrocycle defined by any two adjacent tethers.

To the authors’ knowledge, no “hybrid” ring/atom-bridgehead molecules of the type **XVII** (Figure 9, bottom) have been shown to undergo homeomorphic isomerization. However, Pascal has synthesized compounds with this connectivity, as exemplified by **5** in Figure 10.^33^ Here the bridgehead ring is linked via three short, conformationally restricted tethers to a bridgehead phosphorus atom, for which the lone pair may in principle be *in* or *out*. The synthetic route yielded *in*-**5**, but the tethers were too short/rigid to support homeomorphic isomerization. However, as noted above trivalent phosphorus usually undergoes pyramidal inversion at elevated temperatures, and *out*-**5** could be obtained at 185 °C, as evidenced by trapping experiments. Nonetheless, it can be anticipated that the homeomorphic alternative, which will furthermore exchange the faces of the trisubstituted benzene ring (undetectable in Figure 10), will be realized with suitable higher homologs in the future.

**Figure 10:**
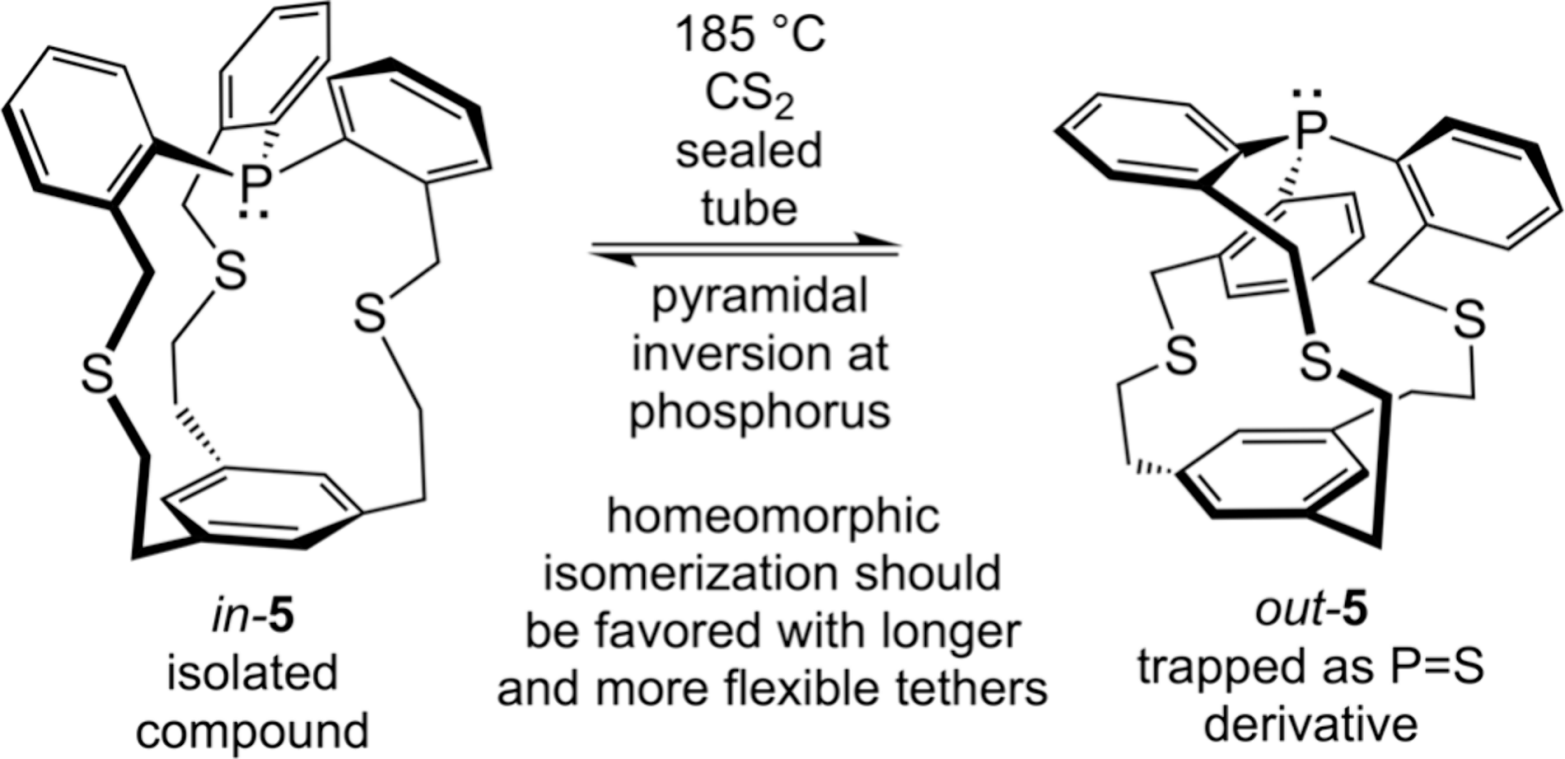
Isomerization of compounds of the types **XVII** (Figure 9), but by a non-homeomorphic mechanism.

There are a large number of macrobicyclic peptides that exhibit hybrid ring/atom bridgeheads analogous to those of **XVII** or **5**. As shown in panel A of Figure 11, this is due to the extensive development of condensations of linear peptides with three pendant -NH or -SH groups with cyclic tris­(electrophiles), as well as transition-metal-catalyzed variants.^12b,34^ Examples include BT8009 in Figure 1 ^14^ and the compounds in panels B–D of Figure 11.^12b,23a,35^ They represent excellent candidates for observable homeomorphic isomers. Surprisingly, the *in*/*out* dispositions of the CH bridgeheads, which would be assayed from the H–C–ring_centroid_ angle, remain undefined in all of these systems.

**Figure 11:**
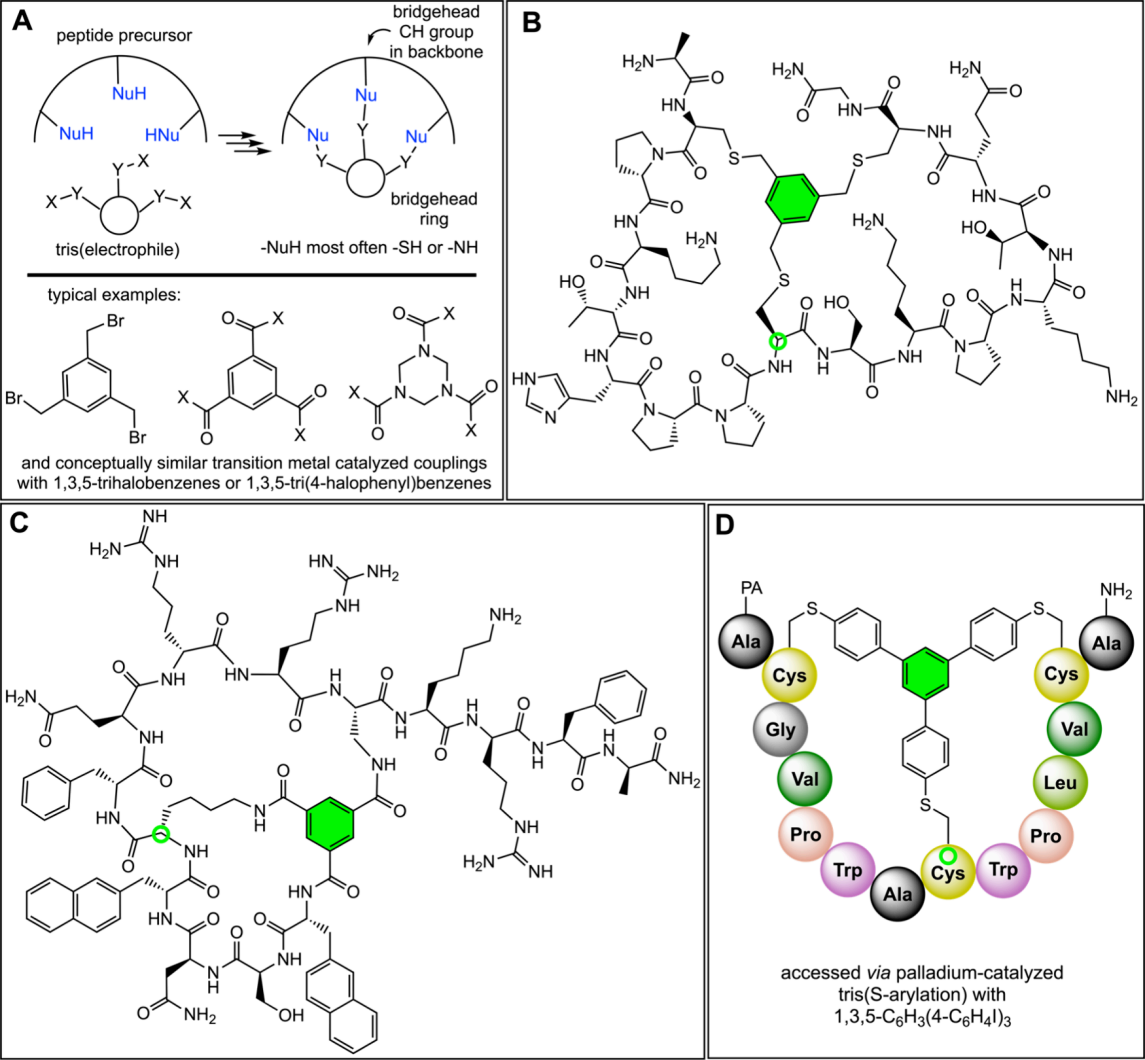
Representative syntheses (panel A) and examples of macrobicyclic peptides that feature hybrid ring/atom bridgeheads (panels B,^12b^ C,^35^ and D^23a^).

## 6 Homeomorphic Isomerization of a Peptide-like Macrobicycle: Implications and Opportunities

A landmark study by Kilburn, seemingly unknown to many in the macrobicyclic peptide community, ties together several themes treated above.^26^ His team synthesized the peptide-like macrobicycle **7** shown in Figure 12 as a mixture of all four isomers—*in,in*, *out,out*, *in,out*, and *out,in*. These could be separated chromatographically at room temperature. The *in,out* and *out,in* isomers are not degenerate, and equilibrated slightly above room temperature (or very slowly at room temperature; *t*_1/2_ 158 h, 20 °C, DMSO). Two of the tethers connecting the bridgehead CH groups consist of 11 atoms, and the third (with the pyridine ring) 15 atoms. These observations indicate that separable homeomorphic isomers are certain to exist for a range of macrobicyclic peptides.

**Figure 12:**
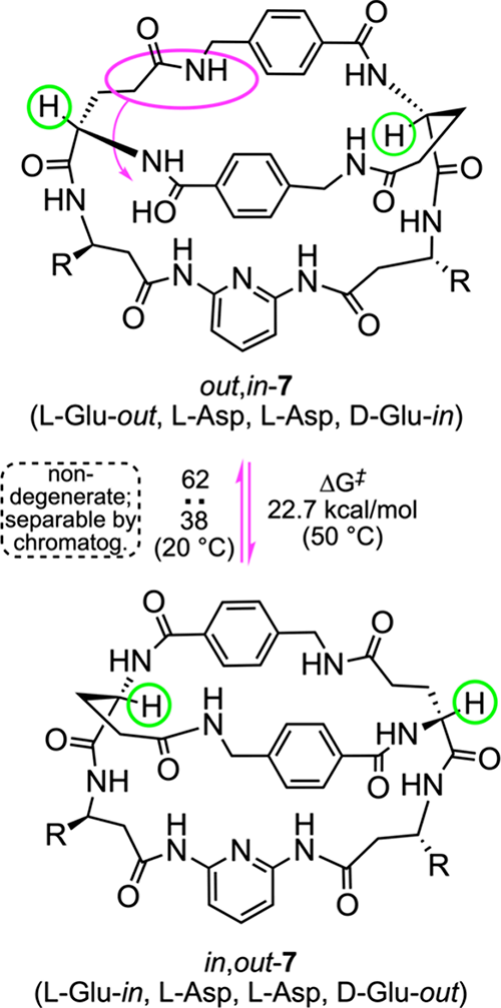
Homeomorphic isomerization of a peptide-like macrobicycle.

Kilburn’s example belongs to the first of the following limiting situations:

1. Two homeomorphs interconvert with a moderate barrier and at room temperature (*t*_1/2_ ∼ 24 h).
  1.1. They may be separately synthesized by stereospecific routes or prepared as a mixture and subsequently separated.
  1.2. Homeomorphic phenomena can be exploited in applications.
2. Two homeomorphs interconvert with an extremely high barrier (much above room temperature).
  2.1. They may be separately synthesized by stereospecific routes or prepared as a mixture and subsequently separated.
  2.2. Homeomorphic phenomena will be challenging to exploit in applications (elevated temperature required, *if* there is sufficient thermal stability).
3. Two homeomorphs interconvert with a very low barrier and below room temperature.
  3.1. Although it will be problematic to separate them, it may be possible to characterize some individual properties or isomerization rates by low temperature techniques (e.g., NMR).
  3.2. Certain homeomorphic phenomena can be exploited in applications (e.g., the U-tube separation in Figure 8).

Three similar limits have been defined for atropisomers,^36^ which as most commonly represented in the literature arise from restricted rotation about a single bond (typically between two appropriately substituted aryl groups).^37^ They have been applied as pharmaceutical and agricultural chemical agents in both mixed and separated forms.

Some examples where the second limit applies have been characterized in the course of synthesizing macrobicyclic peptides related to α-amanitin (Figure 1; see specific case illustrated below).^13b,13c^ Of course, various control elements can be bought into play to alter rates or equilibria of homeomorphic isomerization. For example, regulation could be provided by a photoswitch. Azo (-N=N-) linkages see frequent use, and have in fact been incorporated into macrobicyclic peptides.^38^ Metal ions or other species capable of binding to amide (N^...^C^...^O) linkages should also influence rates and equilibria.

The equilibrium in Figure 12 suggests an intriguing strategy for the stealth oral delivery of a macrobicyclic peptide drug to a cellular target. First, one homeomorph would be designed for the optimal cellular-level therapeutic response. Second, the other would be optimized to survive the gastrointestinal tract and pass through to the epithelia (*vide supra*). One of several approaches would be to administer the drug as the homeomorph that best survives the gastrointestinal tract, but with the homeomorph that best binds the biological target and effects the desired response engineered to dominate at equilibrium.^39^ The first homeomorph would be designed to gradually isomerize at body temperature.

There is an immense literature involving the *de novo* design of cyclic and macrobicyclic peptide drugs that could be applied toward such ends.^5a,40^ These include a variety of sophisticated computational techniques meant to evaluate many thousands of drug candidates. Libraries consisting of thousands of monocyclic^5b^ or bicyclic^41^ peptides have been synthesized and screened. However, to the authors’ knowledge none of these efforts have probed the consequences of homeomorphic isomerization, or even seem aware of the concept.

As an aside, the structures of a number of adducts of macrobicyclic peptides and proteins can be found in crystallographic databases.^42^ Although the algorithm for defining *in* and *out* bridgeheads is simple to apply (**IX**, Figure 2), no assignments have been reported. Of special interest is the *in*/*out* disposition of a free macrobicyclic peptide versus a protein adduct. The authors’ team is in the process of analyzing available data. For example, crystal structures of amanitin (see Figure 1) have been reported for both unbound^43^ and bound states (RNA polymerase II).^44^ The former is for the ß form (ethanol/water solvate), and the latter for the α form, which differs by a OH/NH_2_ or aspartic acid/asparagine substitution. As exemplified for the latter in Figure 13, both exhibit *out,out* geometries, as reflected by C_bridgehead_···C_bridgehead_···H angles of 134.4° and 154.2° (bound) and 129.8° and 150.9° (unbound). It will also be of interest to see to what extent chain crossing occurs in protein adducts (see **IV**, **VI**, **VIII** in Figure 2). To date, such species have been rare^19,20^ but are easily assigned due to their distinctive torsion angle^45^ patterns.

**Figure 13:**
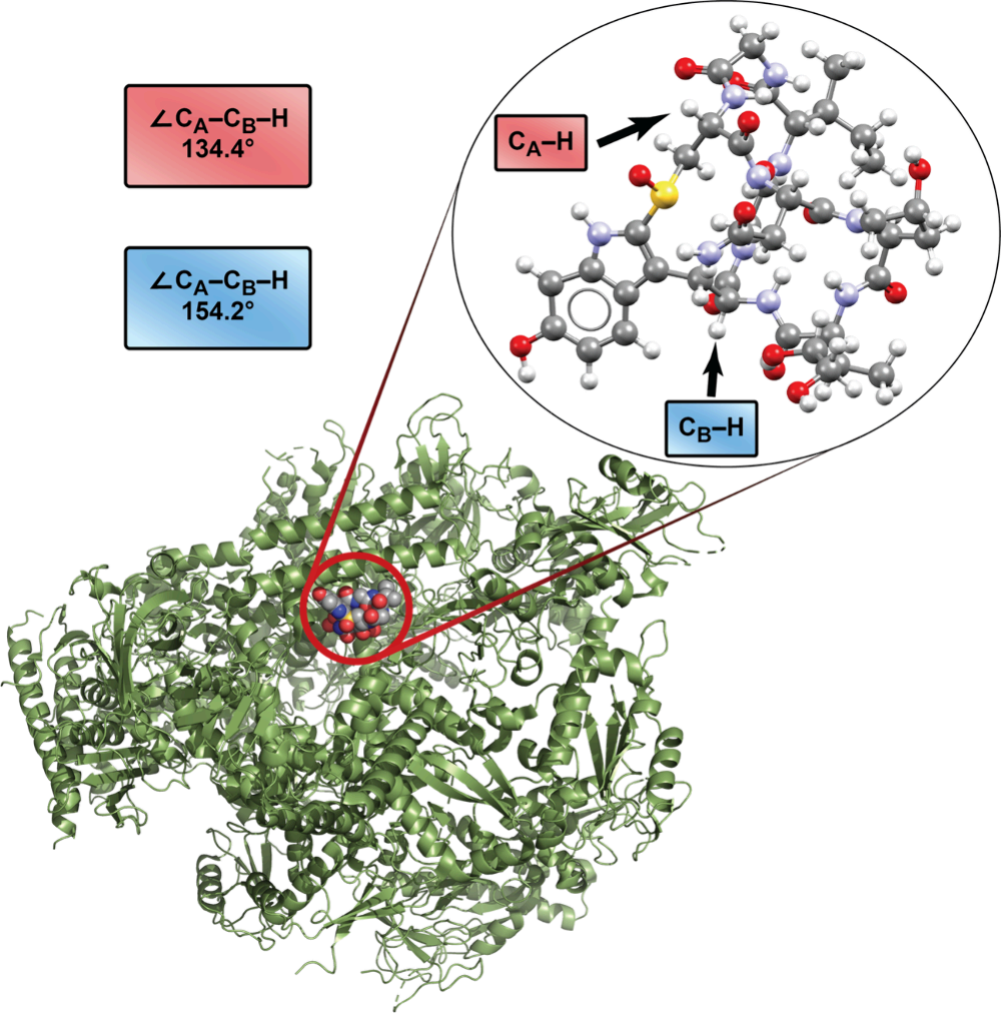
Structure of the adduct of α-amanitin and RNA polymerase II, an *out,out* isomer.

## 7 Macrobicyclic Peptide Drugs: Caveats

Many medicinal agents are chiral, and often only one enantiomer elicits the desired biological response. Scientists in the pharmaceutical industry are acutely aware of the “chiral switch” strategy^46^ by which (1) the organization holding the patent on a racemic drug effectively extends market exclusivity by a subsequent patent involving the enantiopure drug or (2) a competitor organization circumvents the patent on a racemic drug by patenting the enantiopure drug.

Presumably, this would extend to any macrobicyclic drug with tethers of suitable lengths and flexibilities to support homeomorphic isomerization. Thus, it would be possible in the execution of a patent to overlook the possibility of homeomorphs (much as the absolute configuration of a chiral drug was overlooked in many older patents), leaving room for a competitor to follow up with a claim on a specific isomer. Even if a specific form is claimed (e.g., a macrobicyclic drug that crystallized *in,in*), it would be risky not to attempt the investigation of the opposite homeomorph. The patents examined by the authors ignore this issue,^29,48^ and it is inferred that essentially all do.

Some chiral drugs have been patented and marketed as mixtures of enantiopure diastereomers. The “chiral switch” appellation has also been applied when patents for single-diastereomer formulations have subsequently issued.^46c^ As noted above, the compounds in Figure 5 are likely obtained as mixtures of diastereomers at the trivalent arsenic or bismuth stereocenters. These normally have very high barriers to pyramidal inversion, so physical separation or diastereospecific syntheses should be feasible.^49^ As shown in Figure s4, the HPLC data for one bismuth system exhibits a second peak (ca. 10%), apparently due to a species of the same mass as the main peak.^22^ In any case, whatever patented IP may exist for these compounds seems to be silent on these points, leading to vulnerabilities.

Another phenomenon that bedevils intellectual property involving pharmaceutical and agricultural chemicals is polymorphism,^47^ or the accessibility of multiple crystalline forms. In many cases, one modification is superior for reasons of solubility, shelf stability, etc. Rigorously speaking, polymorphs must be comprised of the exact same molecule, and they usually involve alternative conformations (that give rise to alternative crystal lattices). However, crystals may also differ with respect to solvent incorporation, sometimes termed pseudopolymorphism. All of these scenarios can allow end-runs around fine chemical patents.^47^

In the authors’ studies of macrobicyclic compounds, several instances of polymorphism have been encountered. The most instructive for present purposes involves the dibridgehead diarsine **9a** in Figure 14.^22^ This compound can be isolated as an *in*,*in* or *out*,*out* isomer. Each homeomorph can be selectively crystallized (and *out,out*-**9a** crystallizes much faster from *in,in*/*out,out* mixtures). In solution, they slowly equilibrate to a ca. 50:50 mixture. Furthermore, several of the diphosphorus compounds **2** in Figure 4 crystallize in a less stable *out,out* conformation (the thermodynamic preference for *in,in*-**2b**–**e** has been attributed to dispersion forces).^18,21^ The overarching lesson is that macrobicyclic molecules can crystallize in many ways, including a less stable isomer or conformation, complicating the optimum formulation of a claim.

**Figure 14:**
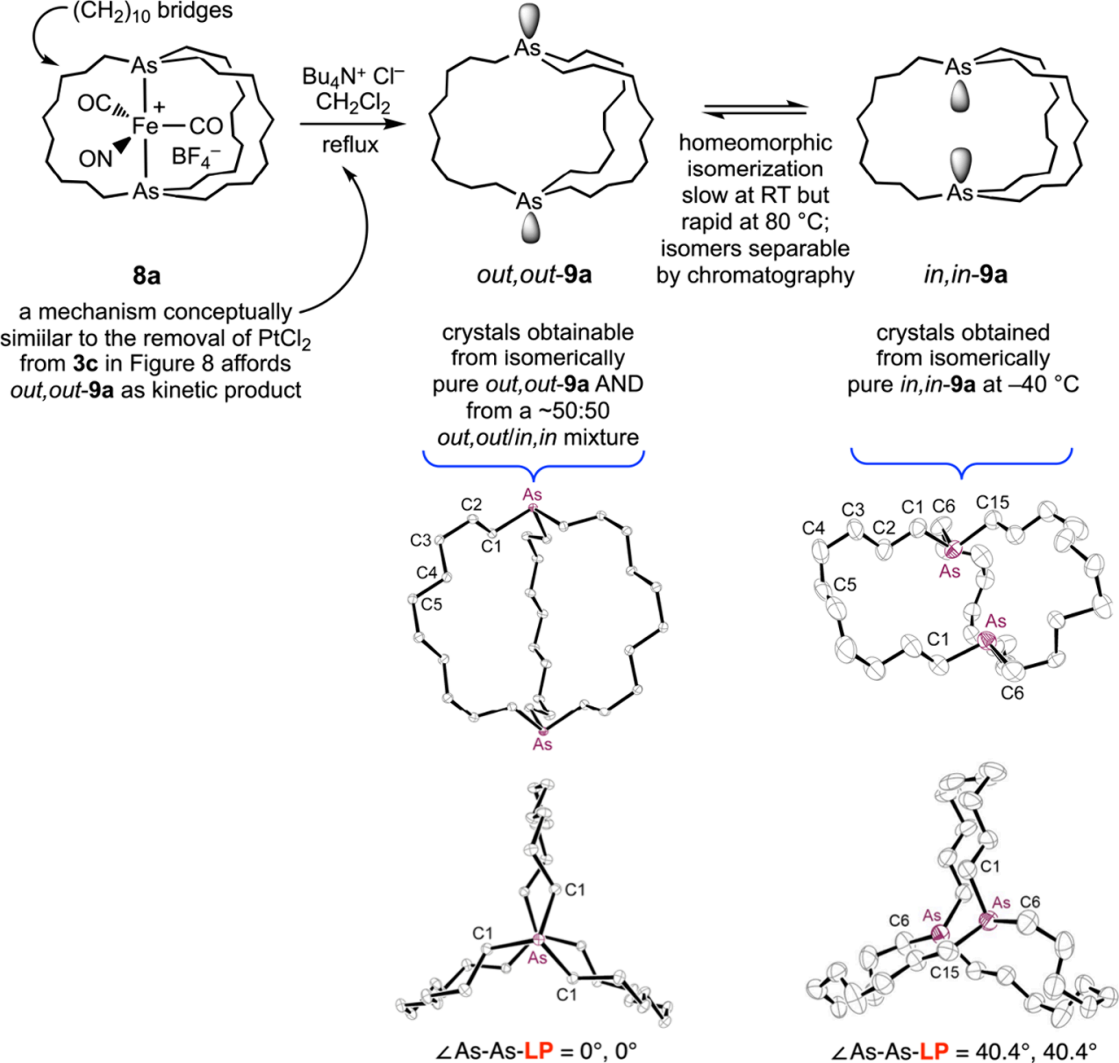
Polymorphs related by a homeomorphic isomerization.

## 8 Related Themes and Topologies: Lasso and Macro*poly*cyclic Peptides

Lasso peptides are a well-established class of macro*mono*cyclic natural products that are attracting great attention.^50^ They feature lasso-like structures as exemplified by **XIX** and **XX** in Figure 15, and only a single total synthesis has been reported to date.^51^ The main challenge is inserting the spoke into the macrocycle. There are several fascinating stereochemical and topological aspects of lasso peptides, as well as one interesting connection to the preceding macrobicycles. Namely, as shown in Figure 15, a lasso molecule is conceptually related to the crossed chain species encountered in homeomorphic isomerization. With very bulky EX groups, the *out,out* adduct **VIII** (see also in Figure 2) can be engineered to be the ground state.^19^ The selective cleavage of the crossed chain near either terminus will generate the constitutionally isomeric lasso molecules **XIX** and **XX**. This represents a rational new approach to the non-enzymatic synthesis of such molecules.

**Figure 15:**
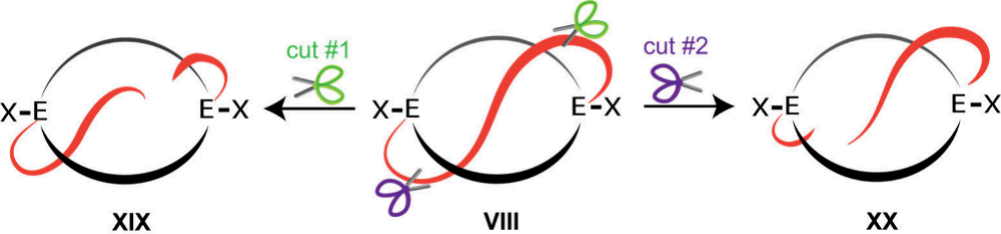
Hypothetical route to isomeric lasso peptides via “crossed chain” *out,out* species.

Throughout this Perspective, reference has been made to studies of additional aspects of macrobicyclic peptide conformations.^13^ Certain isomeric forms, most representing still-small families, have been described as “non-canonical atropisomers”,^13b,37^ “ansamers”,^13c^ and “akamptisomers”,^13a,37^ among other terminology. These are generally applied in the second of three limits defined above—homeomorphs that interconvert only with an extremely high barrier, far above room temperature if at all. Said differently, these can refer to structural subsets that could, in principle, turn inside-out but at a cost of “unphysical” or infeasible bond length stretches (and multiple torsional sequences).

Pascal’s isomers *in*-**5** and *out*-**5** (Figure 10) provide non-peptide examples where interconversion is thus blocked. An instructive peptide example would be the amaninamide isomers **10** in Figure 16, which could be independently synthesized and crystallographically characterized per the structures in Figure s5.^13c^ No interconversion was detected at 150 °C in DMSO, and a CIP-based protocol was devised to differentiate them (*P/M*). Alternatively, as shown in Figure s5 and Figure 16, they can also be represented as *out,out* and *in,in* homeomorphs. In any event, the continued refinement of the concepts and treatments detailed herein and by others will be necessary to (1) describe and/or classify these diverse macrobicycles in the most rigorous, general, and intuitive ways and (2) unravel the isomer space associated with frequently labyrinthine macro*poly*cyclic peptides.

**Figure 16:**
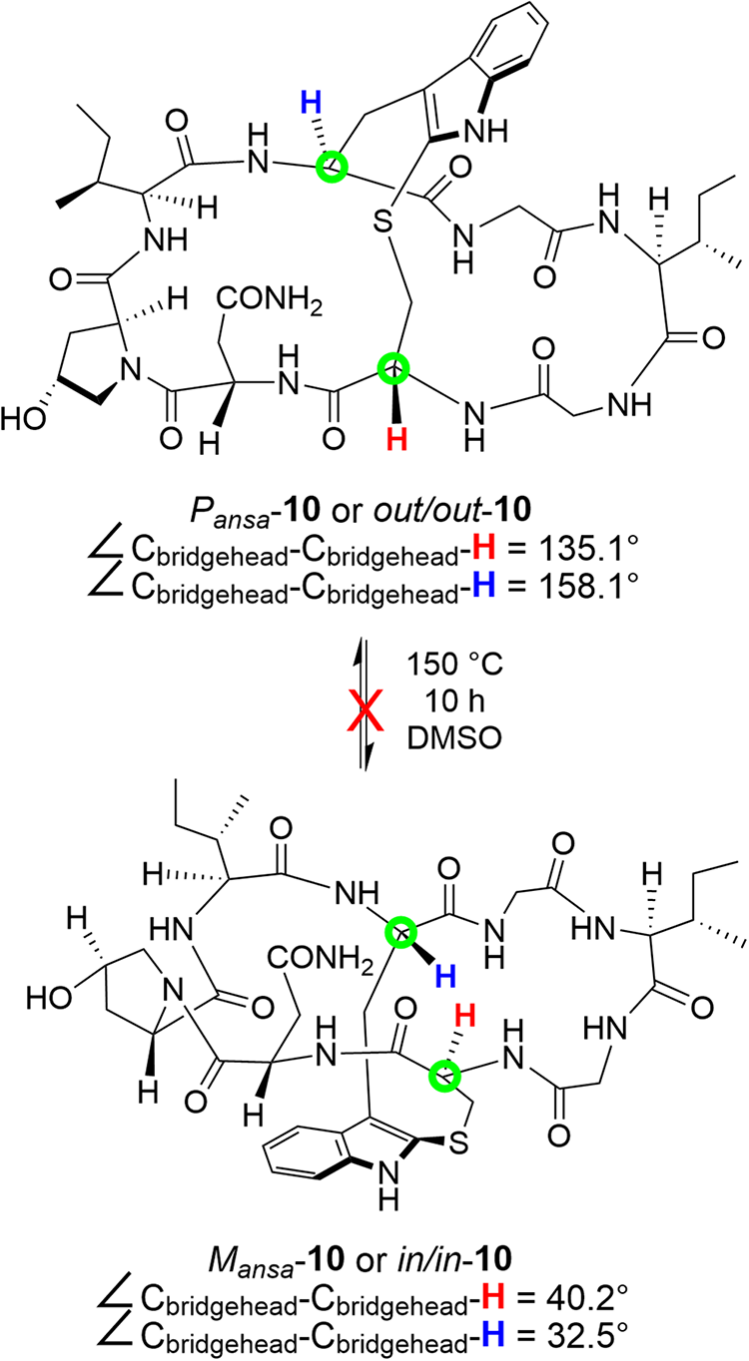
Crystallographically established structures of homeomorphs of amaninamide and angles that allow *in,out* bridgeheads to be assigned (see also Figure s5).

A macropolycyclic system that has passed phase III clinical trials and as of this writing awaits FDA approval, MK-0616, is depicted in Figure 17. This PCSK9 inhibitor has been developed by Merck for the oral treatment of hypercholesterolemia and atherosclerosis.^52^ It consists of two bridgehead rings (5- and 27-membered; shown in red) connected by three tethers (1, 9, and 18 skeletal atoms long). Although it is doubtful that this molecule can easily undergo the type of bridgehead ring/ring homeomorphic isomerization shown for **XVa** and **XVb** (Figure 9), it is certainly the case that distinct homeomorphs may (as with the amaninamides in Figure 16) be accessible.

**Figure 17:**
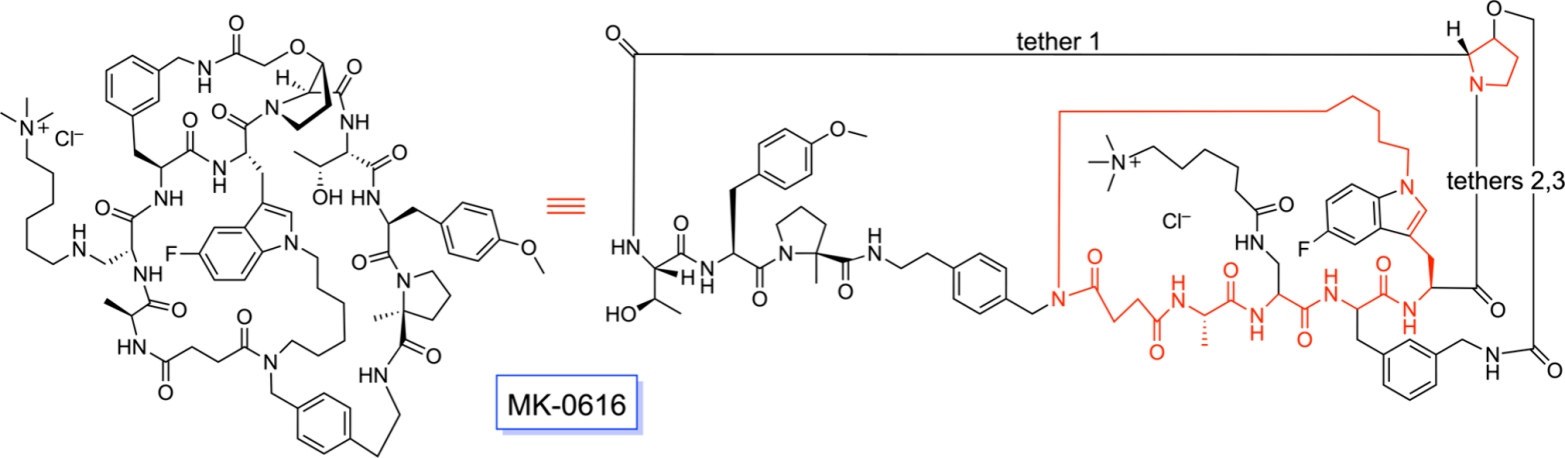
Structural representations of MK-0616, an inhibitor of PCSK9, illustrating the two “bridgehead rings” (red, right) connected by three tethers (cf. **XV**, Figure 9).

## 9 Conclusion

This Perspective has attempted to bridge a classic “two cultures” chasm, integrating concepts from chemistry and biology that involve scientific communities that often have little contact with each other. It is hoped that the concept of homeomorphism can be moved front and center into the canon of macrobicyclic peptide (1) synthesis, (2) characterization, (3) protein and cellular target binding, and (4) biological and medical applications, and furthermore receive consideration as a possible factor in protein misfolding disorders. Without attention to this property, which affects function and efficacy, macrobicyclic peptides and related molecules cannot be considered completely described or characterized. The definition of bridgehead atoms as *in* or *out* deserves particular attention (e.g., Figures 2, 12, and 16), as do the growing number of cases where the absolute configuration of a bridgehead atom is not even established (Figures 5 and s4). Ultimately, homeomorphism should be seen as the basis for innovative and leverageable new design strategies for a wide variety of molecular functions and not an inconvenient detail championed by a small cadre of stereochemistry aficionados.

## Supplementary Material



## Notes

Further graphics illustrating stereochemical features of macrobicyclic molecules, and literature HPLC and MS data consistent with the presence of isomeric macrobicyclic peptides (PDF)
