# Supplementary material for: Overlooked Complications and Opportunities in the Development of Drugs Based upon Macrobicyclic Peptides: The “Homeomorphic Switch”
Source: J Am Chem Soc. 2026 Feb 26;148(9):9156–68. doi: 10.1021/jacs.5c21032 (PMC12983299; doi:10.1021/jacs.5c21032)
Supplement: Supplementary file 1 [file ja5c21032_si_001.pdf]

SUPPORTING INFORMATION FOR

# Overlooked Complications and Opportunities in the Development of Drugs based upon Macrobicyclic Peptides; the "Homeomorphic Switch"

**Isabelle J. Smith, Simon M. Popovic, and John A. Gladysz***

Department of Chemistry, Texas A&M University, PO Box 30012, College Station, Texas 77842-3012, USA.

E-mail: gladysz@mail.chem.tamu.edu

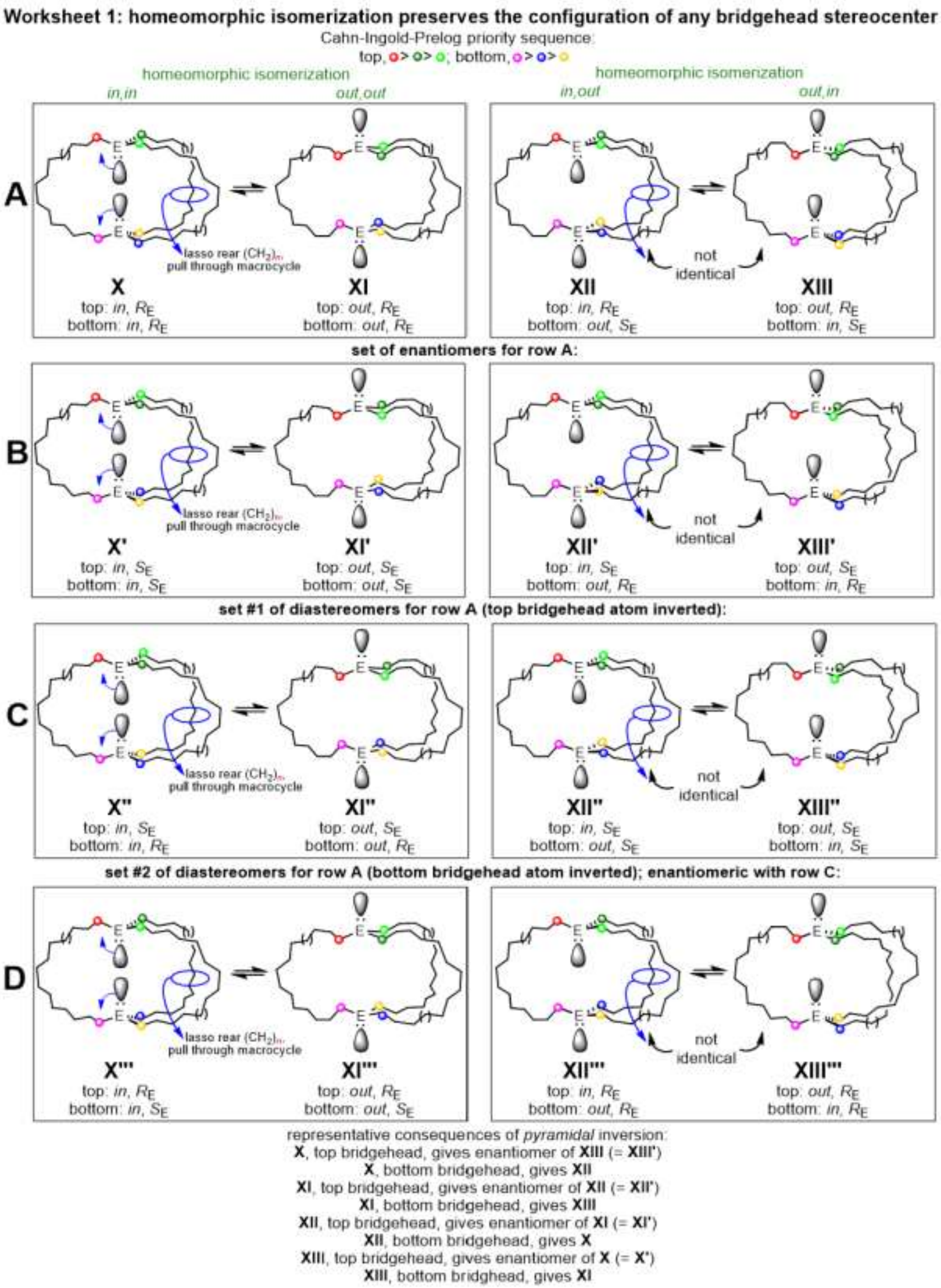


**Fig. s1.** Worksheet showing that in chiral-at-bridgehead macrobicyclic compounds, the configurations of the bridgehead stereocenters are retained upon homeomorphic isomerization, and other stereochemical features.

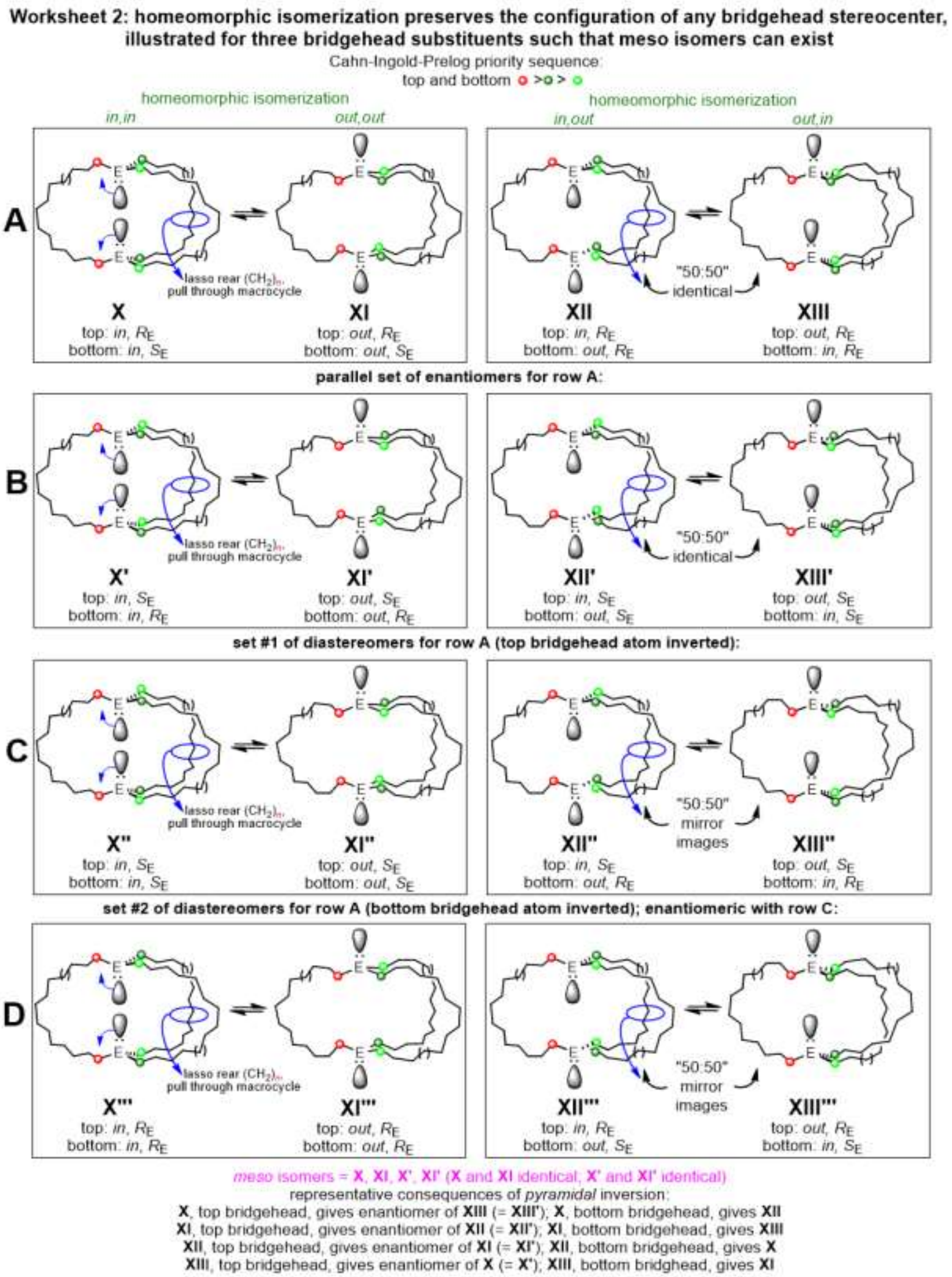


**Fig. s2.** Additional worksheet showing that in chiral-at-bridgehead macrobicyclic compounds, the configurations of the bridgehead stereocenters are retained upon homeomorphic isomerization, and other stereochemical features.

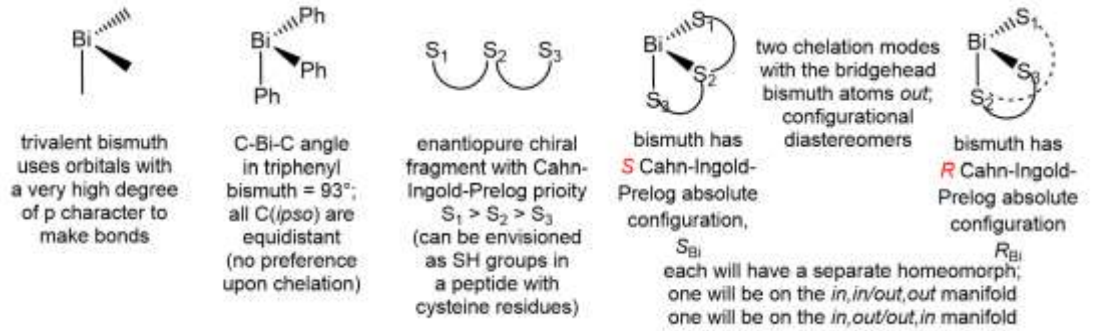


**Fig. s3.** Schematic: generation of diastereomeric macrobicyclic peptides with bridgehead bismuth atoms.

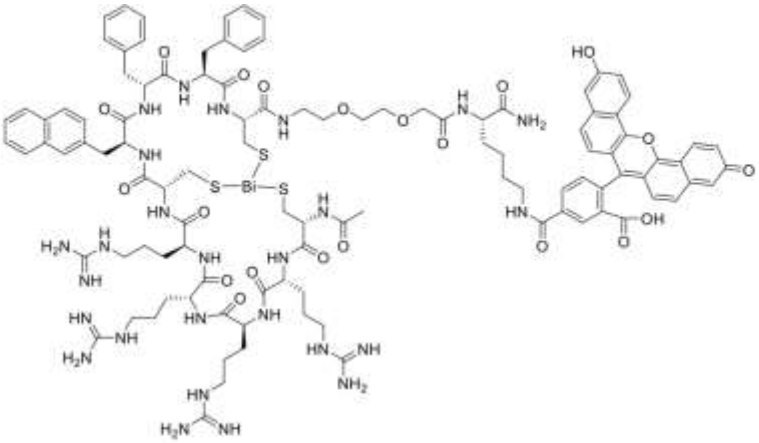

Purity assessment by UPLC (214 nm):

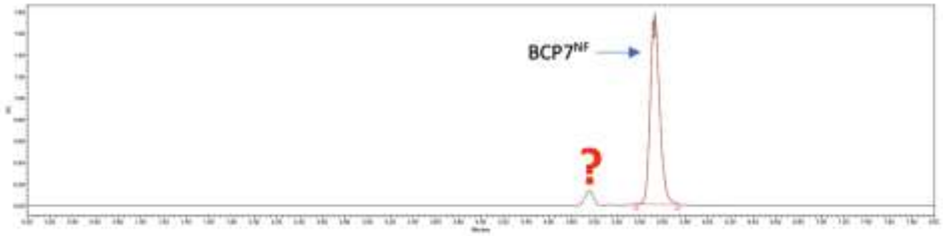


MALDI-MS:

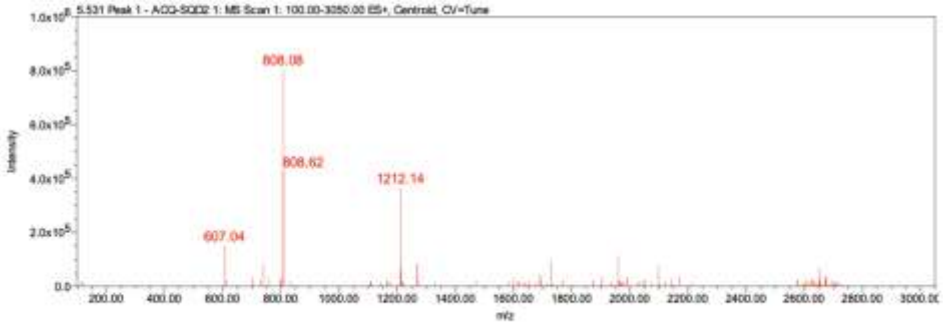


Calculated $[M+4H]^{4+}$: 606.22; observed 607.04
Calculated $[M+3H]^{3+}$: 807.96; observed 808.08
Calculated $[M+2H]^{2+}$: 1211.44; observed 1212.14

**Fig. s4.** Unexplained peak in purity assay for a macrobicyclic peptide with a bridgehead bismuth atom from Ritchey, J. L.; Filippi, L.; Ballard, D.; Pei, D. Bismuth-Cyclized Cell-Penetrating Peptides. *Molecular Pharmaceutics* **2024**, *21*, 5255-5260.

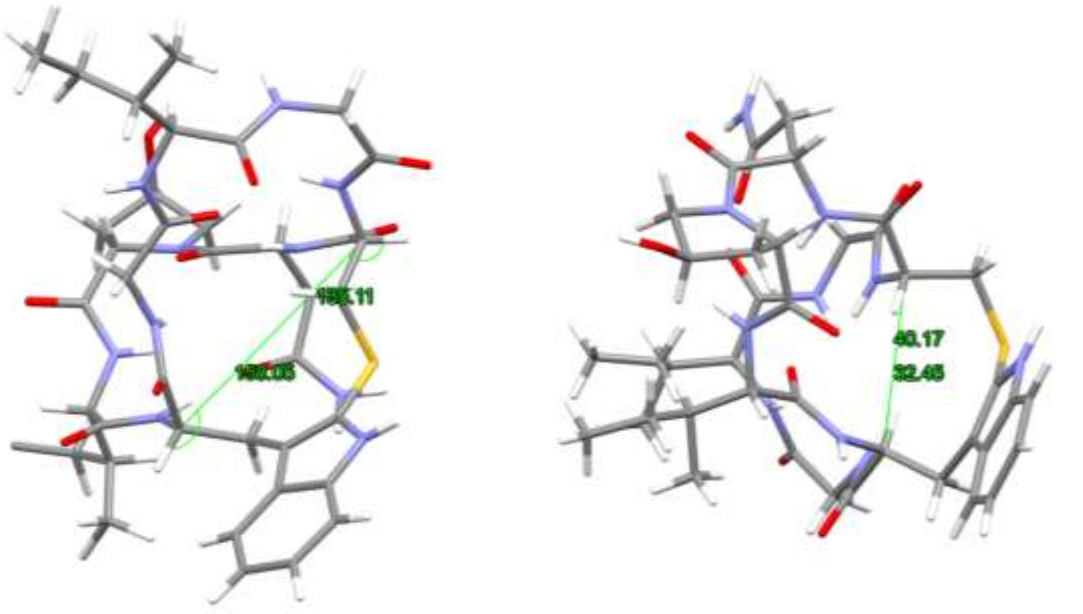

$P_{ansa}$-**10** or *out/out*-**10** $M_{ansa}$-**10** or *in/in*-**10**

**Fig. s5.** Outtakes from the crystal structures of the macrobicyclic peptides in Fig. 16, illustrating the $C_{bridgehead}\cdots C_{bridgehead}\cdots H$ angles.
